# The rise of data repositories in materials chemistry

**DOI:** 10.1038/s42004-024-01143-0

**Published:** 2024-03-22

**Authors:** Konstantin Stracke, Jack D. Evans

**Affiliations:** School of Physics, Chemistry and Earth Sciences, The University of Adelaide, North Terrace, Adelaide, 5005 SA Australia

**Keywords:** Materials chemistry, Materials science, Nanoscience and technology

## Abstract

FAIR (findable, accessible, interoperable and reusable) data practices are necessary to expedite knowledge discovery, encourage collaboration, and optimise resource use, fostering a robust foundation for future scientific progress. Here, the authors explore the use of FAIR practices to advance materials chemistry research, examining key repositories, highlighting their role in sharing scientific data, and examining the accessibility of these approaches.

Modern science is built upon the foundations of previous research, as noted on the current front page of Google Scholar ‘Stand on the shoulders of giants’^1^. To ensure that today’s science is not lost and can be easily built upon by future generations there is a push for the deposition of data in repositories, external to publisher’s constraints on supplementary information^2^. The direction of sharing data is towards FAIR data practices which is essential for enhancing the usability and accessibility of research data^3^. The FAIR principles, which stand for Findable, Accessible, Interoperable, and Reusable, provide guidelines to ensure that research data is easily discoverable, accessible, interoperable with other datasets and reusable for future research. Embracing FAIR data practices accelerates knowledge discovery, fosters collaboration, enhances transparency and reproducibility, and optimises resource utilisation, ultimately advancing scientific progress. There are many online data repositories where researchers can deposit data such that they are accessible to the world. A selection of these currently used in materials chemistry, and the wider scientific literature, are listed in Table 1.

**Table 1:** Summary of data repositories in this study

Repository name	Search keyword
GitHub	github.com
Figshare	doi.org/10.6084
Dryad digital repository	doi.org/10.25338
Open Science Framework	doi.org/10.17605
Zenodo	doi.org/10.5281
Science Data Bank	doi.org/10.57760
NOMAD	doi.org/10.17172/NOMAD
Materials Cloud	doi.org/materialscloud

These repositories are suggested avenues of sharing by an example data science journal^4^ and targeted for general science and materials chemistry. GitHub is a web-based platform crucial for version control and collaborative software development, allowing individuals and teams to manage projects efficiently^5^. It enables the creation of repositories for storing project files, tracking changes, and seamless collaboration through features like branching, pull requests, code reviews, issue tracking and continuous integration, making it a central hub for software developers. Figshare is a web-based platform facilitating the storage, sharing, and dissemination of diverse scholarly content, including datasets, figures, and presentations^6^. Figshare is a centralised repository with digital object identifiers (DOIs) (not generated by services like Github) tied to each entry which enables this information to be cited in the same way as articles or preprints. The DOI system is used by most repositories (if not all academic repositories) and offers a structure for consistent identification, handling metadata, facilitating the automated administration of media and even supporting electronic commerce^7^. Zenodo, developed in part by the European Organization for Nuclear Research (CERN), is an open-access digital repository allowing researchers to share and preserve various research outputs with seamless integration into research workflows^8^. Dryad started as a repository focused on evolutionary biology but it expanded over the years, forming partnerships with publishers and organisations to integrate data sharing into scholarly publishing^9^. The Open Science Framework was launched in 2012 by the Center for Open Science and was initially used for the reproducibility of psychology research but since expanded to cancer biology and is now multidisciplinary^10^. ScienceDB, maintained by the Chinese Academy of Sciences, offers data storage, preservation, and sharing services for global researchers, institutions, and journals^11^. In addition to these general repositories there are also those focused for the data generated by materials chemistry. The NOMAD project, initiated in 2014, includes the NOMAD Repository, providing a platform for sharing electronic structure code simulations^12^. Materials Cloud, operated by École polytechnique fédérale de Lausanne (EPFL) and hosted at Swiss National Supercomputing Centre, serves as a platform for sharing resources in computational materials science, offering tools, simulation software, and curated data^13^.

To understand the use of repositories in scientific literature we parsed the text of articles found using the Dimensions database^14^ that contain the keywords (Table 1), which represent parts of the unique web address or DOIs to these repository platforms. Dimensions is an innovative research information platform developed by Digital Science and provides a comprehensive view of the global research landscape by aggregating research-related information from various sources. We constrained our search by excluding “patents”, “grants” and “policy documents”. This left a series of different publication types, which at the time of this study, includes “articles”, with more than 110 million publications, “chapters”, with more than 14 million publications, “proceedings”, with more than 7 million publications, “preprints”, with more than 4 million publications. The remaining publication types with less than one million entries, “monograph” and “edited book”, were also included.

Our search for each repository demonstrates the accelerating reference to these data repositories (Fig. 1). The storage of software widely used by many researchers on GitHub is observed by the much larger number of references to “Github.com” than any other repository. However, this may be related to the keyword not being a DOI but web address. The next most referenced is Zenodo followed by Figshare, which are both general academic repositories. The more targeted repositories Open Science Framework and Science Databank are both widely referenced, where as the other repositories have less than 100 references in 2023. Zenodo and Figshare are the most widely referenced data-specific repositories, according to this analysis, and the reference of software via GitHub is ubiquitous in the literature. Recently based on push from the community, publishers and funding agencies it is perhaps unsurprising there is an accelerating growth in the use of data repositories. This growth began as early as 2014, as evidenced by our analysis presented here. The data referenced by these publications is broad including computer code used to generate data^15^, gene sequences^16^ and crystal structures^17^.

**Fig. 1:**
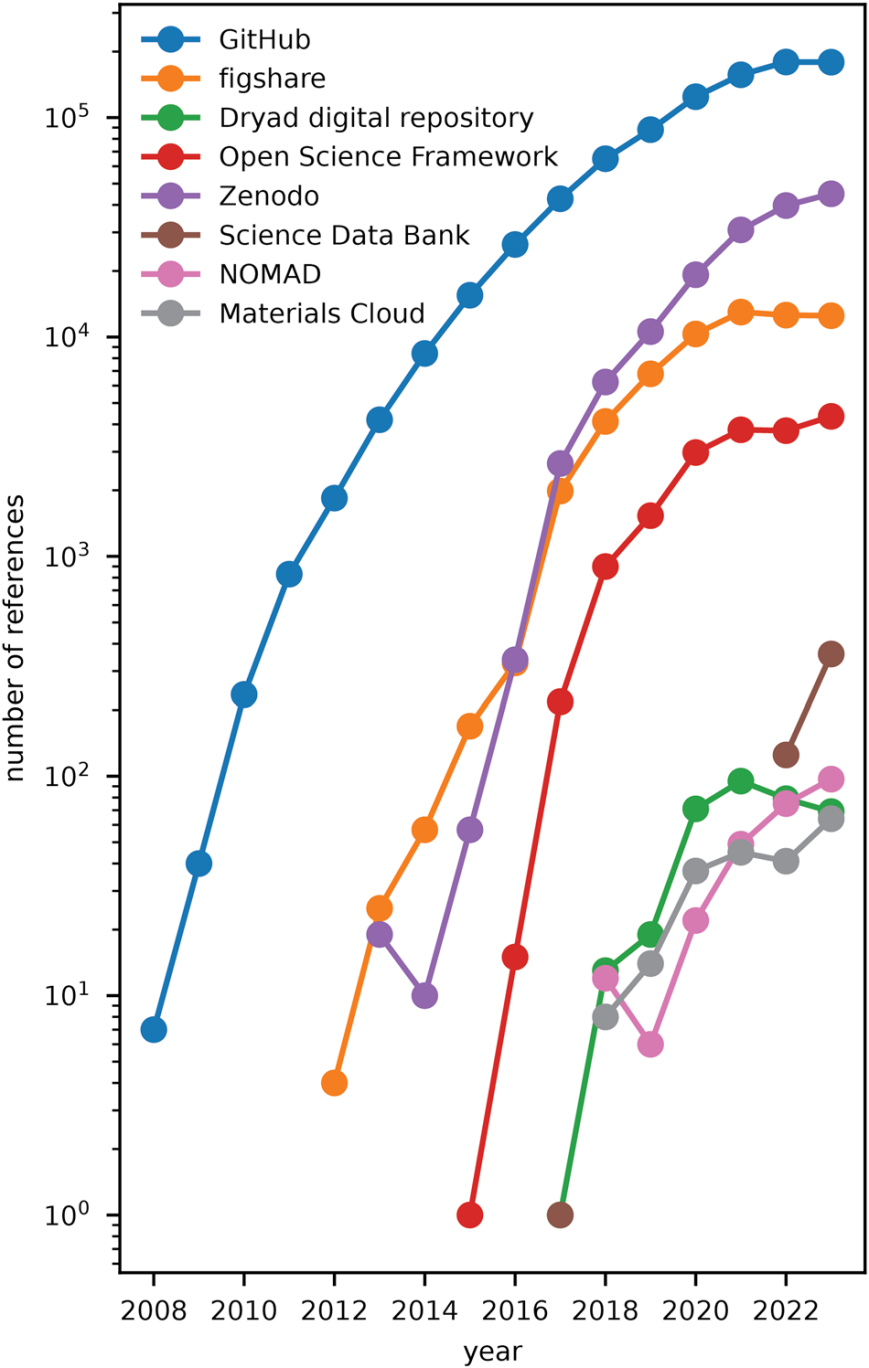
Number of publications that reference each repository since 2008. The number of publications that reference data repositories have increased significantly.

The use of the data shared in these different repositories is domain-specific as some repositories are built to handle specific datasets and adopted by distinct fields. To explore the prevalence of repositories in different scientific fields we evaluated the number of publications referencing each of repositories by research category (Fig. 2). Dimensions uses the fields of research codes set by the Australian and New Zealand Standard Research Classification (ANZSRC 2020)^18^ to label these categories. Our analysis demonstrates that almost 50% of GitHub references are published in the field of Information and Computing Sciences and the Dryad digital repository is mostly used by the Biological Sciences. Unsurprisingly the repositories developed for materials chemistry (NOMAD and Materials Cloud) are referenced in the majority by the Chemical Sciences followed by Physical Sciences. Overall the proportion of the general databases such as Figshare and Zenodo are mostly shared across domains but we note a bias for Figshare references in the Biological Sciences.

**Fig. 2:**
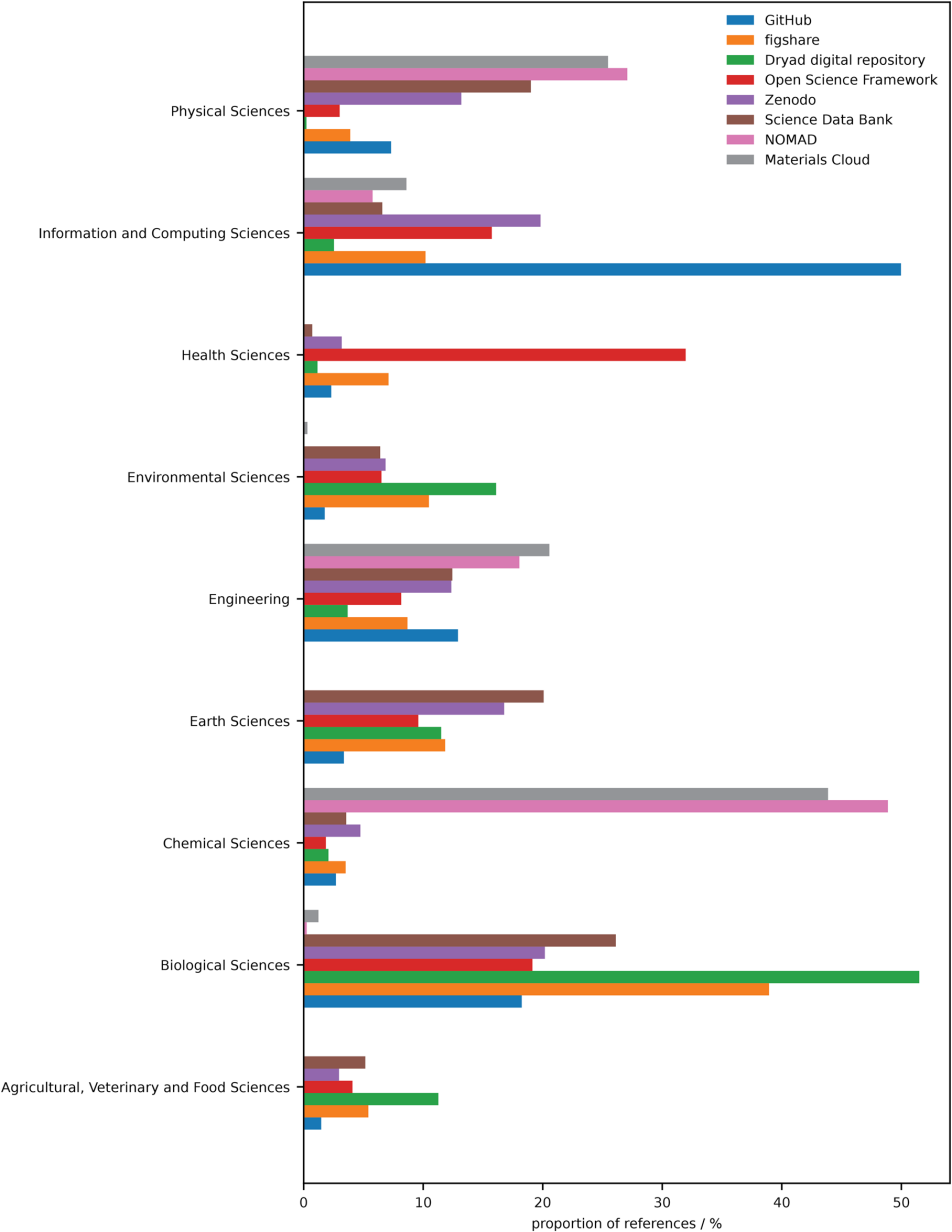
References to data repositories for different research fields. Proportion of reference to each repository categorised by publications associated with fields of research codes set by the Australian and New Zealand Standard Research Classification (ANZSRC 2020).

One complication of using external repositories is whether this information remains accessible over a long time span. To examine the long-term accessibility of data in these repositories, 10 random papers, which referenced the repository and were published within the first years of release, were collected and the links to the repositories were tested if they remain accessible today (Supplementary Tables 1–8). This limited dataset demonstrates that mostly all these references remain active despite ten years passing since some of these repositories were created (Fig. 3). There are protections of provenance that apply to DOI codes (DOIs are static and will not change once being assigned) used by the all services except for Github, which may result in problems referencing software solely stored on Github, however, this does not appear to be a widespread problem. Most, and for some repositories all, of the references we tested resolved to give the information originally referenced. We surmise the broken links found for these repositories that use DOIs are likely due to misprinting errors.

**Fig. 3:**
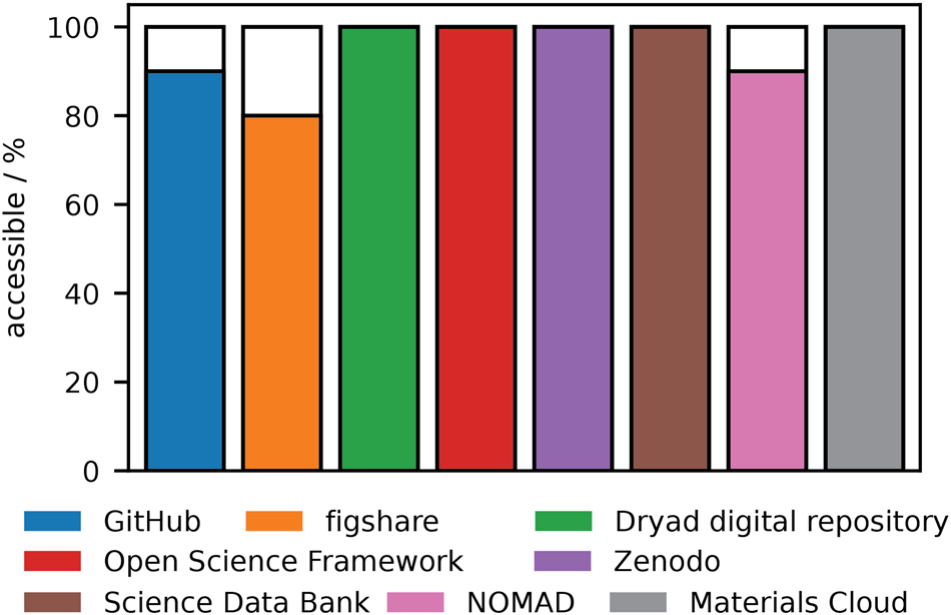
Long-term accessibility of references to data repositories. Proportion of accessible references (that correctly resolve) from 10 random publications for each repository selected from the first years of references.

As discussed by van de Sandt et al.^19^ there is currently no common citation standard for repositories, for example they report only 0.33% of all DOIs registered by Zenodo were cited, and as accompanying articles are usually cited over datasets there is an observed dilution of citations. Despite efforts to implement data citation principles, tracking the impact of data and software based on individual citation counts (in the same way as articles) remains problematic obscuring the understanding of how these data are used in the community. This is obvious by considering one of the most cited chemical science datasets on Zenodo, which contains a large-scale COVID-19 Twitter chatter dataset^20^, currently has 17 citations whereas the accompanying article has more than 100^21^. The community should be encouraged to cite datasets and software present in these repositories as this exactly describes the information used.

The future is bright for the open reporting of data to achieve FAIR data practices. There appears to be an accelerating uptake of the open reporting of science through the use of these external repositories such as Github, Zenodo and Figshare. This approach permits the referencing of datasets and software outside of the constraints often placed by publishers on supplementary information. However, many repositories do have constraints that may limit sharing of specific data types (Zenodo has maximum file sizes of 50GB) and the latest groundbreaking research uses large data sizes. For example research using artificial intelligence in chemistry opens exciting new possibilities^22^ and these approaches employ large language models that can be greater than 100GB^23^, which may necessitate new ways of referencing and depositing information. There is also a concern whether these approaches will stand the test of time and remain active for future generations but we found most of these references are operational, albeit on a limited dataset. We note, however, there are some concerns with respect to data and software shared using GitHub but efforts such as GitHub Arctic Code Vault^24^ may mitigate data loss. Nevertheless, our analysis leads us to expect to see more links to data repositories in the scientific literature going forward.

### Supplementary information

Supplementary Information

## Data Availability

Detailed lists of articles used for testing accessibility and raw data used for analysis were deposited here and at https://zenodo.org/doi/10.5281/zenodo.10704089.
