# Supplementary material for: The rise of data repositories in materials chemistry
Source: Commun Chem. 2024 Mar 22;7:63. doi: 10.1038/s42004-024-01143-0 (PMC10959999; doi:10.1038/s42004-024-01143-0)
Supplement: Supplementary file 1 — Supplementary Information [file 42004_2024_1143_MOESM1_ESM.pdf]

Supplementary Information for: The rise of data repositories in materials chemistry

Konstantin Stracke[1] and Jack D. Evans[1*]

[1*]School of Physics, Chemistry and Earth Sciences, The University of Adelaide, North Terrace, Adelaide, 5005, South Australia, Australia.

*Corresponding author(s). E-mail(s): j.evans@adelaide.edu.au;

Supplementary Table 1 List of DOIs for the tested publications that reference the Github repository. The references consist of 'https://github.com/', which precedes the keyword in the table. The year refers to the year of publication and not the year of data provision on the repository, they may differ. Accessed on the 25/01/2024.

publication	searched reference	year	accessible
10.1021/pr800970z	mcwbbc/vipdac/tree/master	2009	True
10.1093/bioinformatics/btp300	semin/ulla	2009	True
10.1186/1471-2105-10-279	egonw/xws-taverna/tree/master	2009	True
10.1093/hmg/ddp519	bwallace/copd_db	2009	True
10.1145/1394042.1394046	axiom/tree/master	2008	False
10.48550/arXiv.0810.2412	jlaragonvera/Geometric-Algebra	2008	True
10.1007/978-3-642-02032-2_30	jdmunro	2009	True
10.1145/1596638.1596645	jyp/topics/tree/master/	2009	True
10.1109/MIC.2009.74	davebryson/beepbeep/tree/master	2009	True
10.1109/CSE.2009.366	alexy/life-language/tree/master/	2009	True

Supplementary Table 2 List of DOIs for the tested publications that reference the Figshare repository. The references consist of 'http://dx.doi.org/10.6084/m9.figshare.', which precedes the keyword in the table. The year refers to the year of publication and not the year of data provision on the repository, they may differ. Accessed on the 25/01/2024.

publication	searched reference	year	accessible
10.1021/jo401316a	777752<br>779754<br>676012<br>778538	2013	True
10.12688/f1000research.2-58.v2	157057<br>157058<br>157059<br>157060	2013	True
10.12688/f1000research.2-184.v1	783889	2013	True
10.12688/f1000research.2-6.v1	103765<br>103766<br>103767<br>103768<br>103769<br>103770<br>103771<br>103772<br>103773<br>103774<br>103775<br>103776<br>103777	2013	True
10.12688/f1000research.2-138.v2	709569	2013	True
10.1128/genomea.01001-13	830514	2013	True
10.1371/journal.pone.0074770	715895	2013	True
10.1371/journal.pone.0074770	715895<br>715257	2013	True
10.1017/S0031182013001121	102049	2013	False
10.1371/journal.pone.0069741	95635	2013	False

Supplementary Table 3 List of DOIs for the tested publications that reference the Dryad repository. The references consist of 'http://dx.doi.org/10.25338.', which precedes the keyword in the table. The year refers to the year of publication and not the year of data provision on the repository, they may differ. Accessed on the 25/01/2024.

publication	searched reference	year	accessible
10.5194/acp-18-10985-2018	B84K5G	2018	True
10.1371/journal.pone.0195570	B8P88G	2018	True
10.1016/j.compag.2017.09.011	B8WC70	2018	True
10.1016/j.compag.2017.09.011	B8WC70	2017	True
10.5194/acp-18-14585-2018	B8ZK5X	2018	True
10.1016/j.biomaterials.2018.10.024	B8RK52	2018	True
10.5194/acp-18-12141-2018	B8JP4V	2018	True
10.1073/pnas.1718211115	B88593	2018	True
10.3389/fpls.2018.00586	B8CS3Z	2018	True
10.1002/2017WR021977	B8QG63	2018	True

Supplementary Table 4 List of DOIs for the tested publications that reference the Open Science Framework repository. The references consists of 'http://dx.doi.org/10.17605/OSF.IO/', which precedes the keyword in the table. The year refers to the year of publication and not the year of data provision on the repository, they may differ. Accessed on the 25/01/2024.

publication	searched reference	year	accessible
10.1371/journal.pone.0167475	B6KJZ	2016	True
10.7554/eLife.18566	V4CEH	2016	True
10.1016/j.jmr.2015.11.006	K8ZVM	2016	True
10.1371/journal.pone.0149281	3GRW9	2016	True
10.1371/journal.pone.0154696	VKCPM	2016	True
10.1080/09296174.2018.1499457	ZHUJF	2018	True
10.1017/S0033291717001258	MH3CF	2017	True
10.1016/j.dib.2017.08.030	5EZCB	2017	True
10.1080/00224545.2016.1140117	W24DM	2016	True
10.1111/infa.12127	B6G4E	2016	True

Supplementary Table 5 List of DOIs for the tested publications that reference the Zenodo repository. The references consist of 'http://dx.doi.org/10.5281/zenodo.', which precedes the keyword in the table. The year refers to the year of publication and not the year of data provision on the repository, they may differ. Accessed on the 25/01/2024.

publication	searched reference	year	accessible
10.3389/fncom.2014.00157	12798	2014	True
10.12688/f1000research.2-288.v3	8652	2014	True
10.1007/s11852-014-0356-6	1090687	2014	True
10.12688/f1000research.2-184.v1	7099	2013	True
10.12688/f1000research.3-36.v2	8418	2014	True
10.1088/1748-9326/9/11/114017	11395<br>12251	2014	True
10.1038/ncomms6024	10679	2014	True
10.7717/peerj.918	13030<br>13159	2014	True
10.12688/f1000research.12130.2	829949	2017	True
10.1016/j.jtbi.2016.05.001	47152	2016	True

Supplementary Table 6 List of DOIs for the tested publications that reference the Science Data Bank repository. The references consist of 'https://doi.org/10.57760/sciencedb.', which precedes the keyword in the table. The year refers to the year of publication and not the year of data provision on the repository, they may differ. Accessed on the 25/01/2024.

publication	searched reference	year	accessible
10.1016/j.envpol.2022.120185	01723	2022	True
10.1016/j.isci.2022.105658	06259	2022	True
10.1371/journal.pone.0268570	01741<br>01742<br>01743<br>01744<br>01764	2022	True
10.1038/s41467-022-34472-6	02730<br>02764	2022	True
10.1038/s41597-022-01843-z	01872	2022	True
10.1371/journal.pone.0275287	00453	2022	True
10.1080/23802359.2022.2107461	01748	2022	True
10.1016/j.celrep.2022.111290	02073	2022	True
10.1007/s12144-022-04027-5	00063	2022	True
10.1016/j.dib.2022.108420	01694	2022	True

Supplementary Table 7 List of DOIs for the tested publications that reference the NOMAD repository. The references consist of 'http://dx.doi.org/10.17172/NOMAD/', which precedes the keyword in the table. The year refers to the year of publication and not the year of data provision on the repository, they may differ. For the last checked reference the link consist of 'http://data.nomad-coe.eu/raw-data/data/' and the searched reference keyword. Accessed on the 25/01/2024.

publication	searched reference	year	accessible
10.1088/1367-2630/aace6d	2018.02.16-1	2018	True
10.1038/s41524-018-0096-5	2018.06.12-1<br>2018.06.12-2	2018	True
10.1063/1.5037159	2018.07.17-1<br>2018.07.17-2	2018	True
10.1016/j.cpc.2017.02.001	2017.02.19-1	2017	True
10.1103/PhysRevLett.121.146401	2018.09.21-1	2018	True
10.1088/1361-648X/aa7ca7	2017.06.30-1	2017	True
10.1038/s41467-018-02918-5	2017.11.10-1	2018	True
10.1038/s42005-018-0009-4	2018.01.03-1	2018	True
10.1088/1367-2630/aa57c2	2016.11.02-1	2017	True
10.1038/s41467-018-05169-6	2018.05.28-1<br>Rnh/Rnh_4DFTJQgTSOib4e4d-5GByiTVB.zip<br>R10/R10ncY1AZG6X9y-Nj8F0_DiN8NeLD.zip<br>RsL/RsLoZhSAdK0BopfI2T4B5pLfMyjVN.zip<br>RMG/RMGpPc3B_HiR0D-oLE4ND66HmYdH-.zip<br>Re2/Re2mnhOAs6ZNqvTY1p-W2RavinjOM.zip<br>R9u/R9usAWjw2xq9F8zW-66jyCyeDLlDa.zip<br>Rkx/RkxmUCgPxt-9xDdIpr5xqPQK8PC9H.zip<br>Rdz/RdzeezGR0W5wGEpGYEqOq7AygYS9J.zip<br>Rc_/Rc_XxYadb0ZlfBVLqCNo-EtVocxv8.zip<br>RKX/RKXqE9xPCiLlufNK0n4pbtzdbID5H.zip<br>Ryv/RyvdvBLf1QdM5QJ_8DVve7CknkdK5.zip<br>Rut/Rut3qcReY6SJO6fIJ5jangTSlMjaQ.zip<br>Reg/Reg0D-KojGnrw51EYl2Q2rCYOIfJM.zip<br>RSk/RSkoltrNkpZwp1xpi_Oj1jO4IndC5.zip<br>RA-/RA-tqhSlH5idfPW_3UxE80I7BBL6s.zip<br>Ray/RayT1o-XjyZaWdlVS_Fk8nssdO1w9.zip<br>Rlb/RLbbgx7klbZ7ZdO5O_YABQGjBOZ9g.zip<br>RcC/RcC8TDWGWCtQLhWeB2a1N8y9Q7y4r.zip<br>Ra8/Ra8nAuJOgxGwSytw1scU5BTeB3ozo.zip	2018	True<br>False

Supplementary Table 8 List of DOIs for the tested publications that reference the Materials Cloud repository. The references consists of 'https://doi.org/ 10.24435/materialscloud:', which precedes the keyword in the table. The year refers to the year of publication and not the year of data provision on the repository, they may differ. Accessed on the 25/01/2024.

publication	searched reference	year	accessible
10.1021/acs.chemmater.8b01425	2018.0003/v2	2018	True
10.1038/s41597-019-0080-z	2019.0015/v1	2019	True
10.1021/acscentsci.8b00157	2018.0004/v1	2018	True
10.1073/pnas.1805062115	2018.0013/v1	2018	True
10.1038/s41598-019-53815-w	2019.0042/v2	2019	True
10.1038/s41467-019-08483-9	2018.0011/v3	2019	True
10.1038/s41524-019-0151-x	2018.0019/v1	2019	True
10.1038/s41597-019-0157-8	2019.0002/v2	2019	True
10.1038/s41467-018-04618-6	2018.0010/v1	2018	True
10.1038/s41467-019-10663-6	2019.0017/v1	2019	True
